# Form and Law ‐ Rupert Riedl's Significance for Morphology

**DOI:** 10.1002/jez.b.70030

**Published:** 2026-06-14

**Authors:** Michael Schmitt

**Affiliations:** ^1^ Universität Greifswald, Zoologisches Institut und Museum Greifswald Germany

**Keywords:** burden, explanation, homology, morphological laws, Popper

## Abstract

Rupert Riedl showed in his “Order in Living Organisms” that morphology can produce law statements and is, therefore, a proper, that is, nomothetic, science. Furthermore, he coined useful terms (interphene and metaphene) and concepts (burden, cadre and minimal homology). Notwithstanding certain flaws—he used “laws in evolution” and “verification of a theory” although claiming to be in accordance with Popper's hypothetico‐deductivism—and an almost complete absence of synecological relationships in his evolutionary explanations, his scientific legacy still forms a basis of a “logic of morphology” and an “evolutionary morphology,” especially through the works of his students.

## 1 Introduction

Rupert Riedl (22 February, 1925–18 September, 2005) neither discovered or described an important morphological structure that was then named after him, nor did he develop a useful technique or method for morphological research, and he worked himself morphologically only within the framework of taxonomic studies, e.g. in *Fauna und Flora der Adria* (Riedl 1963). Thus, it needs a justification in the first place why he could be named a “liberator of morphology” (Schmitt 2007). Actually, the relevance of Riedl's contribution to morphology can only be understood when considering the political and economic situation in Central Europe in the 1970s and the philosophical setting of the majority of scientists (and laypeople) at that time. Although Karl Popper's hypothetico‐deductivism was long published (original German edition “Logik der Forschung” 1934/5, English edition “Logic of Scientific Discovery” 1959) and widely accepted in principal, the traditional “naïve” philosophical approach was still virulent in many, if not the most, academic teachers of the time. This way of thinking is based on the idea that there is an increasing degree of reliability and verisimilitude from an initial idea over hypothesis–theory–regularity–rule–principle–to law. The concept of “science” in the German‐speaking community differs crucially from that in the anglophone world. The German term “Wissenschaft” is not a synonym of the English “science”. “Wissenschaft” comprises “Naturwissenschaft” (roughly corresponding to “science”) and “Geisteswissenschaft” (approximately equal to “humanities”). According to Wilhelm Dilthey's basic distinction, “Naturwissenschaft” aims at *explaining* its subject, whereas the topic of “Geisteswissenschaft” is to *understand* its subject (Dilthey 1910). The most used way to explain something is to prove that what is to be explained (explanandum) is a special case of a superordinate regularity, a law. This type of explanation was formalized by Hempel and Oppenheim (1948). Consequently, also morphologists needed laws in order to explain their findings if morphology should be classified as a “Naturwissenschaft” proper. Even Riedl (1983: 206), although a “passionate morphologist“ (Schoch 2010), stated that the method of morphology “is, in principle, hermeneutical,” that is, designed to understand, and “does not … contain the explanatory procedure for the phenomena in these disciplines” [= biosciences].

Wilhelm Windelband (1894) introduced the terms nomothetic (setting laws) versus idiographic (just describing) to characterize the different fields of “Naturwissenschaft” and “Geisteswissenschaft”. As Adolf Meyer (1926, e.g., 84) stated, morphology is purely descriptive, thus not nomothetic, and consequently unable to explain anything according to the Hempel‐Oppenheim scheme. Thus, morphologists in Germany, and probably also elsewhere, found themselves in a deep crisis of legitimacy in the first half of the 1970s. Already in 1967, Wilhelm Schäfer, at that time director of the Senckenberg Museum in Frankfurt am Main, saw—referring to Rudolf Richter's papers of the 1940s – that “The descriptive natural sciences have begun to be ashamed of their name…“ (“Die beschreibenden Naturwissenschaften haben sich ihres Namens zu schämen begonnen…“). Ernst Mayr disparagingly claimed that “morphology is nothing but German idealistic philosophy” in a personal conversation with Rupert Riedl (Riedl 1983: 206). In my own experience in the early 1970s, “students who demanded ‘science in the service of the people,’ but also politicians who questioned the efficiency of university funding, and not least the esteemed colleagues from the ‘application‐oriented’ branches of biology, who were thus in a secure haven, questioned the raison d'être of morphology.” (Studierende, die “Wissenschaft im Dienst des Volkes” forderten, aber auch Politiker, die nach der Effizienz der eingesetzten Hochschulfinanzen fragten, und nicht zuletzt die werten Kollegen aus den “anwendungsnahen” Zweigen der Biologie und damit im sicheren Hort, fragten nach der Daseinsberechtigung der Morphologie) (Schmitt 2007).

In this context, it matters decisively that, beginning with the reformation of the universities by the end of the 1960s, the number of positions for professors in zoology in Germany increased dramatically during the 1970s. However, this increase did hardly affect morphology but was nearly exclusively due to the expansion and novel foundations of the disciplines of physiology—mainly neurophysiology—and ecology (see Wägele and Bode 2007, 17). Morphology, that is, the morphologists, held a weak position in the competition for finances, rooms and staff. Right in this period of crisis, Rupert Riedl's “Ordnung des Lebendigen” appeared (Riedl 1975, English 1978).

### 1.1 Riedl's Contributions

It soon became apparent that this book had a significant influence on the self‐image of morphologists, even if certainly not many read it completely page by page. In a highly elaborated language, Riedl developed step by step a method of reasoning from realizing chances when throwing dice to approaching certain ground for explanation by calculating probabilities of the evolution of complex organismic structures, that is, the predictability of order (Schoch 2010). A central point of his approach is the claim that morphology can be used to formulate universally valid laws. The word “law” is found in high frequency, as the following examples may show:

*“c. Redundancy content and* law *content*

We have already met two of the parameters applicable to redundancy *(a* and *R)*. The number of identical occurrences of a message *(a) is* crucial for the recognition of a determinative occurrence (Section I *Ble). I* have used the redundancy content *R*, on the other hand (Section I *B2a)* to indicate the number of redundant decisions in such a determinative occurrence, assuming provisionally that all these decisions occur (for with systemization, as later shown, it is possible to eliminate many of them). Having defined *R* we can subtract these recurring decisions from the total quantity of information, i.e. from the determinacy content *D*. The remainder *L* corresponds to the content of the original communication or statement, i.e. to the *law* content of a determinative occurrence.

(1) L=D—R

This important entity *L*, which determines all the repetitions of a determinative event, corresponds to the idea of law, conformity to *law* or regularity in ordinary speech. Thus determinacy content of an occurrence, up till now measured in *bitsD*, can be stated in a more differentiated form as *law* content plus redundancy content, i.e. *bitsL + bitsR*. In the same way *L* can be defined from the quotient of the determinacy content *D* and the length of the series or relative redundancy *r* (where *r* = *D/L)*, that is *L = Dir*. It follows that

(2) D=L•r

We can therefore describe determinacy as regularity times the repeated occurrence of decisions. *But*, again, such instances conforming to a *law* are what we understand by order …”. (Riedl 1978, 13)

This example illustrates the complex formalized language of “Order in Living Organisms”, and also the frequent reference to “law” (emphases added by me). What “law” means in this context is defined on 22: “Order is *law* times the number of instances”. And even the term most central to morphology appears as a law: “The *law* of homology, from molecules to behavior” (Riedl 1978, 246, emphases added by me).

Starting from simple considerations about the probability of events occurring, Riedl showed in a stringent manner that living beings are constructed according to a lawful order. The laws governing this order can be identified and can serve as a causal explanation for empirically collected morphological findings. These laws explain why certain morphological structures could not be evolved, while for others there was no morphological alternative in evolution. Of course, like any law of nature, these laws have the logical status of a theory, but nothing less than that.

**Figure 1:**
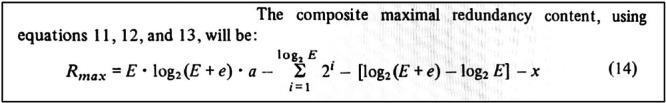
One of Riedl's examples of a mathematical formalization of his line of argument (1978, 16).

Immanuel Kant, arguably the most influential and highly revered philosopher of German tongue, declared in 1786 “…I maintain that in any particular natural science, only as much *actual* science can be found as *mathematics* can be found in it” (Ich behaupte …, daß in jeder besonderen Naturlehre nur so viel *eigentliche* Wissenschaft angetroffen werden könne, als darin *Mathematik* anzutreffen ist—emphases added). This apodictic statement tormented morphologists at the end of the 20th century like a thorn in their side as they had little mathematics to show for themselves in their field of work. Here too, Riedl's approach offered a satisfactory solution to the problem. He had developed numerous equations like the one in Figure 1.

Riedl also formalized his concepts in a proper mathematical fashion, as on page 140:

*“a. Quantitative characteristics”*

In the first place the degree of fixation (F) and degree of burden (B) are so strongly correlated on average that one can be described as a mathematical function of the other:

F = *f (B). As Table A and Fig. 44 indicate, the fixation time for a minimal burden of B* = 1 is F = 10 6. We know this value already for we have met it as*Ps (cf. equation 30*, Section V В 1 h) and also as*P, • Pe (cf. equation 26). 17 Beyond this I now assert that the*

function, if expressed in log — log form, is a straight line such that a change in the burden of three orders of magnitude gives a change in the fixation of two orders of magnitude.

Therefore:

$$F\approx B^{2/3}\times 10^{6}”\left(\text{emphases}\;\text{added}\right)$$

**Figure 2:**
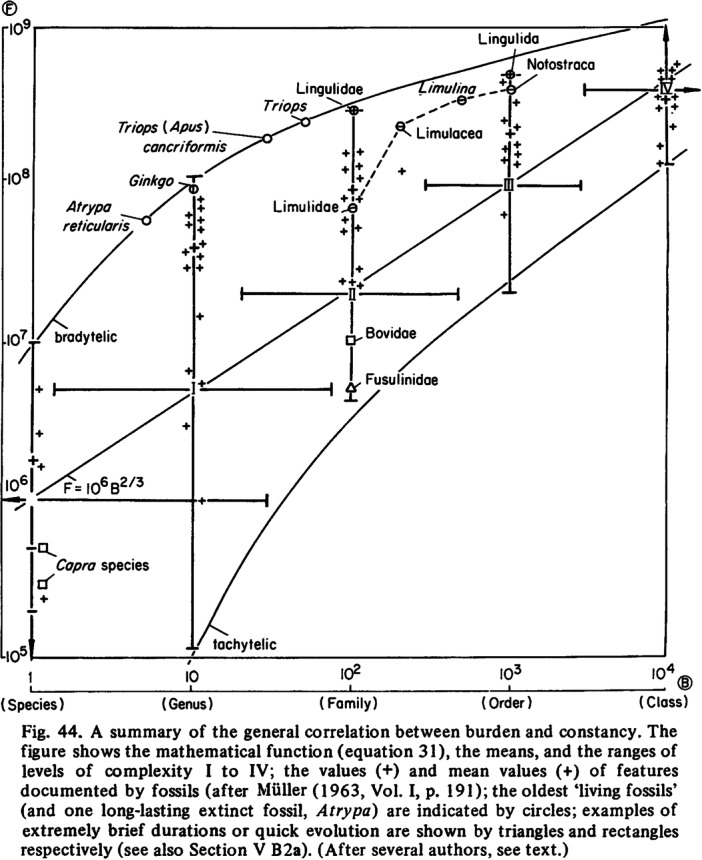
A graph from Riedl (1978) based on morphological data but showing the same design as any graph in the experimental disciplines.

And he presented graphs exactly resembling those in publications in the experimental disciplines. An example is given in Figure 2 (Fig. V54 in 1975, fig. 44 in 1978). The straight line represents the correlation bet

ween the degree of fixation (F on the y axis) and the degree of burden (B on the *x* axis) according to equation 31: F ≈B^2/3^ × 10^6^. The crosses represent actual measured values, taken from various publications and set out in Table A. Riedl distinguishes four “levels of complexity” (also explained in table A), indicated by the Roman numerals in the center of the correlation curve.

In the present context, it does not matter whether or not the scientific content of this graph is relevant, correct, or sensible. I just use this figure to show that Riedl presented morphological data in a mode perfectly resembling figures in papers of experimental branches of biology, and until then highly unusual in morphology. He was certainly strongly influenced by Ludwig von Bertalanffy, one of the four “Gestalten” who had the most important impact on his scientific development (the other three being Konrad Lorenz, Karl von Frisch, and Wilhelm von Marinelli, Riedl 2004, 73–75).

Riedl's difficult‐to‐read book thus represented morphology's entry ticket into the world of nomothetic sciences. Thus, Riedl cured the inferiority complex of morphologists towards the experimental natural sciences.

In “Order in Living Organisms” Riedl demonstrated, convincingly in my view, that and how complex organismic structures are systems. Although he did nowhere provide an explicit definition of “system”, it is evident that he saw them as complex wholes formed by the cohesion and interdependence of their parts. His “systems theory of evolution” explains (1) why and how complex structures could evolve “against all odds”: because “internal selection” eliminates non‐functional traits and supports advantageous steps already early in ontogeny, that is, before the organism interacts with the external environment. And it makes clear (2) that and how established complex traits have only a limited capacity of modification: because all structures carry a certain “burden” (see Figure 2), and the higher the burden, the higher the constancy (for an explanation of “burden” see next paragraph).

### 1.2 Riedl's Valuable Legacy

Even if some philosophical and biological weaknesses in Riedl (1975, 1978) can be found in this book (see below), it nevertheless represents a milestone in the establishment of a modern ‘logic of morphology’. What I refer to below as Riedl's most important contributions is based, admittedly, on an arbitrary evaluation.

- The morphological laws postulated by Riedl make causal explanations possible, according to the Hempel‐Oppenheim scheme (Hempel and Oppenheim 1948).
- He introduced useful terms, for example, “interphene” and “metaphene.” “… definitive phenes can be called metaphеnes. Not until later were these built over by further differentiations and became preliminary stages in development. These preliminary stages can be called intermediate or interphenes for they are certainly identical to the metaphenes of the ancestors” (Riedl 1978, 209). These terms describe in concise manner what plays a major part in the discussion of Haeckel's concept of recapitulation.
- He established the concept of “burden”. This means “the responsibility carried by a feature or decision” (Riedl 1978, 80). The burden can be quantified: “The degree of burden is genetically specified by the number of subsequent decisions that depend on a preliminary decision or by the number of single events (or features) functionally dependent on a preliminary decision or on a fundamental event (or feature)” (104). “This can be illustrated anthropomorphically by thinking of the responsibility with which a feature is burdened. Objectively it corresponds to a systemic position … within the complexity of the system under study. Differences in burden thus range over three to four orders of magnitude. Some burdens are a 1000 or 10,000 times greater than others. ….

Burden does not necessarily depend on the complexity … of the feature itself. It is true that a more complex feature carries somewhat more burden than a less complex one of similar systemic position. Thus in the… example, if the aortic bulb is added to the ascending branch of the aorta then the burden of the coronary arteries is also added …” (129). Although Riedl did not at all deny the importance of selection, i.e. the interaction of organism and environment, he focused on the heavy functional demands within organisms, granting the internal component of selection the predominating role in the evolution of body plans (Schoch 2010). Here, he understood the term “function” to refer mainly to internal functioning and gave less thought to its “ecological role” (Bock and von Wahlert 1965).

An outstanding feature of Riedl's burden concept is its strict emphasis on causality (Schoch 2010). The main effect of burden was that neither development nor function (here: internal function as well as ecological role) could be changed at the same time in the same direction. Thus, burden canalizes ontogenetic development as well as evolutionary trajectory. This has a significant impact on our understanding of body plans, particularly with regard to the question of why there are so few of them (Wagner and Laubichler 2004; Schoch 2010).

- He introduced the distinction between “cadre homologues” and “minimal homologues.” “Cadre homologue[s] …. provide the framework or cadre for further subordinate homologues. In the… example this would be all those homologues from the concept of the vertebral column down to that of the odontoid process (Fig. 11 a‐f in Riedl 1978).

For a vertebral column of middle degree of differentiation the number of cadre homologues can be calculated as follows: 1 inclusive concept (vertebral column); 5 regions in the column; 7 vertebrae per region (on average); 4 main parts of the vertebra per vertebra (on average); 5 subordinate parts of the vertebra per main part (on average) = 1 + 5 + 35 + 140 + 700 = 881 cadre homologues.

Minimal homologue[s… are] all homologues at the bottom end of the hierarchical sequence × in the example the ventral articular face of the odontoid process. They are characterized by the fact that they cannot be further divided into subhomologues in their particular hierarchical sequence. No homologues occur beneath them but only identicalities of a different kind.

…. This lower limit is important because it limits the number of individual homologues in every system and allows them to be counted, given sufficient knowledge” (41).

Riedl's concept of “homology” was further refined and explicated by his student Günter P. Wagner 1989a; 1989b; 1994. Mary Jane West‐Eberhard, for example, drew on these and Riedl's own publications in her highly stimulating — and certainly controversial work “West‐Eberhard (2003).

The concepts of “Burden” and of “Cadre/minimal homologues” proved to be enormously stimulating and fruitful. They allowed for quantifications and conclusive explanations of countless empirical morphological phenomena. Riedl's ideas are crucial elements of other fundamental concepts, even if his works are not always cited but instead those of his students, for example, those by Günter P. Wagner. This is the case, for example, in Graham E. Budd's primer on “Morphospace” (2021) where an important source is Stadler et al. (2001). Riedl's statements on impossible or at least highly improbable morphological structures (263) correspond exactly to the concept of morphospace. Riedl was cited relatively rarely, Google Scholar finds—on 19 May, 2026—870 citations of his 1978 book, as compared to 11759 citations of Gould and Lewontin's 1979 paper on the “spandrels of San Marco (where Riedl 1975 and 1977 are cited). This may be because few of his publications appeared in English, and because his seminal work (1978) is so difficult to read.

Stefan Richter and Christian Wirkner refer explicitly to Riedl's concepts in their “research program for evolutionary morphology” (Richter and Wirkner 2014). They explain in their introduction “A view of morphology as general as the one put forward herein has received repeated but isolated support over the past decades. Advocates include … Riedl in his fundamental works ‘Order in Living Systems’ (Riedl 1975, 1978) and ‘Structures of Complexity’ (Riedl 2000)”.

These examples demonstrate Riedl's importance for morphology as a science. But at least as important is his justification of morphology as a separate way of thinking. He did this in numerous books, only a few of which have been translated into English. We find a characteristic phrase in his chapter on “The role of morphology in the theory of evolution” (Riedl 1983, 237): “We can say: morphology contains the theory by which the internal and systematical conditions of evolution are to be understood, and we describe the conditions of evolution by a theory according to which we understand the a prioris of our morphological preunderstanding.”

These thoughts are elaborated in detail in a small book that is only available in German (Riedl 2006, published posthumously by his daughter Barbara Schweder) “Der Verlust der Morphologie” (The Loss of Morphology). Here, Riedl describes that we humans perceive the world when and because we look and think morphologically. We interpret complex gestalten as a whole, without the need to analyse an item detail by detail. This is an innate ability, acquired in evolution because “it is vitally important to interpret both the distant and the half‐hidden lionizers correctly” (p. 13). Morphology plays also an important part in our world view because we think in “fields of similarity” (Ähnlichkeitsfelder, p. 14). As an explication he describes the process of recognizing an item found in the sand at the beach:

„The handle of a piece of sports equipment seems to be sticking out of the beach; touching it with your shoe reveals a long bone. Immediately, the uninvited sports equipment disappears from the interpretation and, just as uninvited, makes way for all the long bones we may have seen. This approach can be understood from the fact that variations of objects are far more common than absolute uniqueness; in other words, an expectation that helps us find solutions” (Der Griff eines Sportgerätes scheint aus dem Strand zu gucken, die Berührung mit dem Schuh fördert einen Röhrenknochen zu Tage. Sofort verschwinden die ungerufen erschienenen Sportgeräte aus der Deutung und machen, ebenso ungerufen, allen Röhrenknochen Platz, die wir gesehen haben mögen. Diese Anlage ist aus dem Umstand zu verstehen, dass die Abwandlungen von Gegenständen weit häufiger sind als die absoluten Einmaligkeiten; also eine Erwartungshaltung, die uns bei der Lösungsfindung unterstützt).

Further, Riedl emphasizes that nothing can be explained without its context [= cadre] (dass nichts ohne seinen Rahmen zu erklären ist, p. 56). This does not just apply in the field of linguistics where the meaning of a word or a statement can correctly be understood only if the context is known. Riedl means that we reach an understanding of natural items, be they structures or taxa, only within the known context. And it is certainly relevant that the German “Rahmen” not only translates to “context” but also to “cadre”, which relates to the field of homology research.

### 1.3 Critique

Riedl referred repeatedly to Popper's hypothetico‐deductivism (Popper 1934, 1959). Central to this philosophy is the assumption that there are no laws in history (e.g. Popper 1969, 1994: 180) and that science has no chance to decide what is really “true” (e.g. Popper 1974). Nevertheless, Riedl stated (1978, 267) “There is also a general law of tran[s]specific evolution….” as well as that there is the “… law of descent in biology… “ (1978, 275). These are just two examples where Riedl's use of “law” in “Order in Living Organisms” is at odds with Popper. According to Popper's “Critical Rationalism” (or “Hypothetico‐Deductivism”) any assertion that claims validity beyond the observable individual case is a theory, and a theory can never be verified, it can only be tested and then either be falsified or stand the test, that is—in Popper's words—become corroborated. In Rield's book, however, we read, for example, on p. 241. “The explanatory value of a theory … contains one more important feature. That is the possibility of verification.” Even though he occasionally uses the term ‘verification’ in the context of confirming individual predictions, the fact remains that he explicitly claims that his *theory* can and should be verified.

Another point of criticism, albeit more of a weakness, of Riedl's theory is its exclusive focus on the Hempel‐Oppenheim type of explanation. This is especially evident in Riedl's discussion of “features of maximum freedom”, for example, the shape of orchid flowers on pp. 134 ff. (Figs. V25‐31 in the German edition, fig. 39 in the English book). He hardly says a word about the interspecific relationships that most probably are responsible for the evolution of these features. Co‐evolutionary interactions are nowhere mentioned as selective factors in “Order in Living Organisms”. This means, Riedl was either not aware of or purposefully ignored the type of explanation that was termed “historical‐narrative” by Walter J. Bock (1981) but was already introduced substantially by Ernest Nagel in 1961 (there called “genetic explanations”, p. 25 f.). As co‐evolution is a historical process, it cannot be fully understood and explained by general laws, statements of antecedent conditions and logical deduction, that is, through a “nomological‐deductive” explanation. Explaining a synecological relationship as the result of co‐evolution needed, like any explanation of a historical event, to invoke a “historical narrative” argument.

## 2 Conclusion

This attempt to characterize Riedl's significance for morphology represents my personal view and cannot claim to be a professional theoretical analysis. When reading through Riedl's opus magnum, the “Order in Living Organisms”, I found some points to criticize and others to accept and to admire. What I found worthy of criticism is reference to “laws in evolution” and to “verification of a theory” although he claims to follow Popper, and the almost complete absence of the historical‐narrative mode or syn‐ecological interactions in his evolutionary explanations.

What I regard as his most important legacy in morphology is the concept of burden, i.e. “the responsibility carried by a feature or decision” and the introduction of the distinction between cadre and minimum homologies. He established a way of formulating laws that enable quantitative statements to be made in morphology and for causal evolutionary explanations.

Riedl's ideas, especially conveyed through the work of his students, are effective in concepts like “morphospace” and “constraint”. They form an essential foundation for the concept of Evolutionary Morphology, and, last but not the least, they show morphology as a way of thinking. Rupert Riedl teaches us that morphology is not simply another branch of biological science, but rather the heart of organismic biology.

## Data Availability

Data sharing not applicable to this article as no datasets were generated or analysed during the current study.
